# Signature pathway expression of xylose utilization in the genetically engineered industrial yeast *Saccharomyces cerevisiae*

**DOI:** 10.1371/journal.pone.0195633

**Published:** 2018-04-05

**Authors:** Quanzhou Feng, Z. Lewis Liu, Scott A. Weber, Shizhong Li

**Affiliations:** 1 Bioenergy Research Unit, US Department of Agriculture, Agricultural Research Service, National Center for Agricultural Utilization Research, Peoria, IL, United States of America; 2 Institute of New Energy Technology, Tsinghua University, Haidian Qu, Beijing, China; 3 USDA-MOST Joint Research Center for Biofuels, Peoria, IL, United States of America; Hubei University, CHINA

## Abstract

Haploid laboratory strains of *Saccharomyces cerevisiae* are commonly used for genetic engineering to enable their xylose utilization but little is known about the industrial yeast which is often recognized as diploid and as well as haploid and tetraploid. Here we report three unique signature pathway expression patterns and gene interactions in the centre metabolic pathways that signify xylose utilization of genetically engineered industrial yeast *S*. *cerevisiae* NRRL Y-50463, a diploid yeast. Quantitative expression analysis revealed outstanding high levels of constitutive expression of *YXI*, a synthesized yeast codon-optimized xylose isomerase gene integrated into chromosome XV of strain Y-50463. Comparative expression analysis indicated that the *YXI* was necessary to initiate the xylose metabolic pathway along with a set of heterologous xylose transporter and utilization facilitating genes including *XUT4*, *XUT6*, *XKS1* and *XYL2*. The highly activated transketolase and transaldolase genes *TKL1*, *TKL2*, *TAL1* and *NQM1* as well as their complex interactions in the non-oxidative pentose phosphate pathway branch were critical for the serial of sugar transformation to drive the metabolic flow into glycolysis for increased ethanol production. The significantly increased expression of the entire *PRS* gene family facilitates functions of the life cycle and biosynthesis superpathway for the yeast. The outstanding higher levels of constitutive expression of *YXI* and the first insight into the signature pathway expression and the gene interactions in the closely related centre metabolic pathways from the industrial yeast aid continued efforts for development of the next-generation biocatalyst. Our results further suggest the industrial yeast is a desirable delivery vehicle for new strain development for efficient lignocellulose-to-advanced biofuels production.

## Introduction

The industrial yeast *Saccharomyces cerevisiae* is widely applied in starch-based fermentation industries for ethanol production. The native *S*. *cerevisiae* is superb in glucose consumption but limited in uptake and utilization of pentose such as xylose. This has been a major obstacle for efficient cellulosic ethanol production from lignocellulosic materials. Although it is not a natural xylose utilization yeast *S*. *cerevisiae* does pose a pathway for oxidizing xylose [***1–3***]. However, in this pathway, xylose was not recognized as a metabolic fermentation carbon source but led to yeast starvation and respiratory response as observed in recombinant *S*. *cerevisiae* strains [***4, 5***]. Over the past decades a significant advance has been made to improve xylose utilization for *S*. *cerevisiae* with improved ethanol yield ranging from 0.09 to 0.46 g g^-1^ as reviewed elsewhere [***6, 7***]. However, the challenge remains since the limited rate of xylose conversion and ethanol productivity for genetically engineered *S*. *cerevisiae* are not readily for economic industrial applications [***8***].

**Fig 1:**
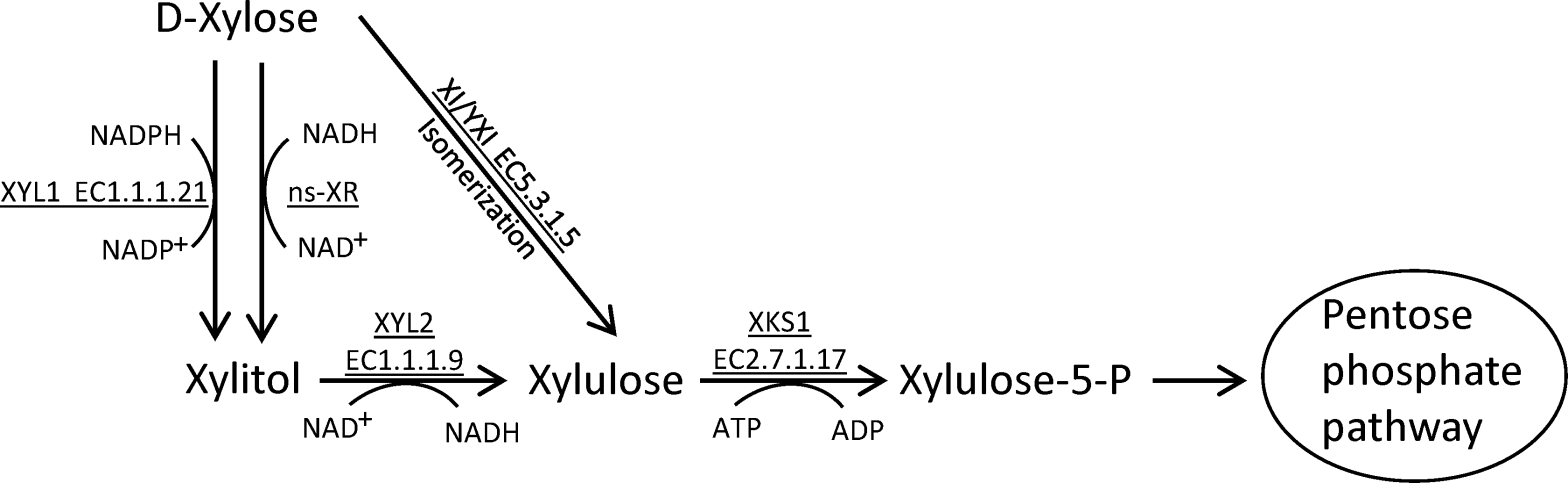
Xylose metabolic pathways. A schematic illustration of typical xylose metabolic pathways applied for genetic engineering of *Saccharomyces cerevisiae*. Enzyme-encoding genes and EC numbers are presented as follows: xylose reductase (XYL1, EC1.1.1.21), non-specific aldose reductase (ns-XR), xylitol dehydrogenase (XYL2, EC1.1.1.9), xylulokinase (XKS1, EC2.7.1.17), and xylose isomerase (XI/YXI, EC5.3.1.5).

Introduction of oxidoreductase reaction pathways from *Scheffersomyces stipitis* is commonly applied to enable *S*. *cerevisiae* utilizing xylose for ethanol production [***9–11***]. In this pathway, xylose is first oxidized into xylitol by xylose reductase (XR, *XYL1*, EC 1.1.1.21), and xylitol is further reduced into xylulose by xylitol dehydrogenase (XDH, *XYL2*, EC 1.1.1.9). Then xylulose is phosphorylated into xylulose-5-phosphate by xylulokinase (*XKS1*, EC 2.7.1.17) prior entering into the pentose phosphate pathway (Fig 1). However, this method causes cofactor imbalance and increased xylitol production as a byproduct [***12, 13***]. In this pathway, the xylose-to-xylitol conversion releases NADP^+^ while the xylitol-to-xylulose reduction reaction yields NADH. When electron acceptor is short under anaerobic conditions, yeast cells are unable to maintain a sound redox balance. Even when a xylose-to-xylitol conversion was coupled with NADH, the higher ratio of NADPH/NADH still led to more xylitol accumulation since such a pathway was relatively weak [***14–16***].

Another popular approach is to use a bacterial pathway applying xylose isomerase gene *XI/xylA* from bacterial *Thermus thermophilus* and *Clostridium phytofermentans* [***17, 18***] or fungal species from *Piromyces* and *Orpinomyces* [***19, 20***]. This pathway initiates xylose metabolism through its isomerization into xylulose by xylose isomerase (*XI*, EC 5.3.1.5) (Fig 1), which avoids cofactor imbalance associated with the xylitol formation and reduction. However, the limited *XI* expression in the yeast often results in lower xylose conversion and ethanol productivity. When grown on mixed sugars of glucose and xylose, the recombinant strains preferred glucose, and the xylose consumption was substantially slow with a low affinity of xylose transportation kinetics [***15, 21***].

Sugar transporter is a necessary first step for carbohydrate utilization. *S*. *cerevisiae* has plenty glucose transporters but lacks an efficient xylose transporter system. In order to improve xylose uptake and utilization for *S*. *cerevisiae*, many heterologous xylose transporter genes were evaluated, including those from *Arabidopsis thanliana*, *Candida intermedia*, *Debaryomyces hansenii*, *Neurospora crassa* and *S*. *stipitis* [***22–27***]. However, most heterologous xylose transporter genes showed poor expression in *S*. *cerevisiae*, and no satisfactory level of improvement was observed on laboratory strains. On the other hand, the industrial yeast strains appeared to have a different response. Using an industrial yeast strain of *S*. *cerevisiae* as a host, overexpression of individual xylose transporter genes from *S*. *stipitis* improved the rate of volumetric xylose consumption [***28***]. A serial of new genotypes of an industrial yeast strain with an individual xylose transporter gene from *S*. *stipitis* increased ethanol production from xylose [***26***]. In a comparative study, all five industrial strains outperformed another five laboratory strains engineered with the same pathway in both xylose consumption rate and ethanol productivity [***29***]. We previously developed a tolerant industrial yeast strain NRRL Y-50049 that is able to *in situ* detoxify major class of toxic chemical compounds liberated from lignocellulose biomass pretreatment such as 2-furaldehyde (furfural) and 5-(hydroxymethyl)-2-furaldehyde (HMF) [***30, 31***]. We further enabled Y-50049 to utilize xylose by genetic engineering and generated strain NRRL Y-50463. Strain Y-50463 contains a synthesized yeast xylose isomerase gene *YXI* in its chromosome XV and a set of plasmid-carried heterologous genes including *XYL2*, *XKS1*, *XUT4* and *XUT6* [***32, 33***]. Strain Y-50463 is able to grow on xylose as its sole carbon source and ferment ethanol on mixed sugars of glucose and xylose in the presence of fermentation inhibitors furfural and HMF [***33***].

Due to the well known genetic background and readily available genetic tools of yeast model strains, laboratory strains were widely used for studies on xylose utilization in *S*. *cerevisiae*. For example, characterization on xylose induced effects against glucose on metabolism and gene expression was reported using traditional XR-XDH pathway for laboratory strains [***34***]. Transcriptome and proteome of a laboratory strain using the same XR-XDH pathway was characterized for cells grown under aerobic batch culture conditions [***35***]. Ethanol fermentation process occurs under anaerobic or oxygen-limited conditions. Characterization of aerobic grown cells aids understanding cell growth response but has limited impact on ethanol fermentation under anaerobic conditions. Recently, a transcriptome analysis of a xylose-utilizing flocculating industrial yeast was reported [***36***]; however, it was again applied the conventional XR-XDH pathway. XI pathway has a significant advantage over the traditional XR-XDH pathway but relatively fewer information is available. Furthermore, single genes were often identified but reports on gene interactions are rare. There is an especial lack of pathway-based knowledge on xylose utilization in XI-pathway for genetically engineered industrial yeast *S*. *cerevisiae*. The advanced development of qRT-PCR technology allowed more accurate quantitative analysis of gene expression that surpasses high throughput method such as microarray. Recently developed pathway-based qRT-PCR array provided an efficient platform for comparative analysis of a subset of genes that suitable for more defined and closely related pathway analysis [***31, 37***]. In this study, we explore the first insight into the important gene interactions in the centre metabolic pathways of the genetically engineered industrial yeast Y-50463 using comparative gene expression analysis. The quantitative expression analysis of Y-50463 on a time-course study revealed a unique signature expression profile of the industrial yeast. Such a signature pathway expression of Y-50463 underlines the pathway-based genetic interactions of the improved xylose utilization for the genetically engineered industrial yeast. Knowledge obtained by this investigation aids continued efforts for the next-generation biocatalyst development for low-cost cellulosic ethanol production.

## Materials and methods

### Yeast strains

An industrial yeast type strain *S*. *cerevisiae* NRRL Y-12632 obtained from ARS Cultural Collection (Peoria, IL USA) was used in this study as a parental strain control. Strain Y-50463 is also known as ATCC 18824, WRI74, CCRC 21447, DBVPG 6173, DSM 70449, IFO 10217, IGC 4455, JCM 7255 and NCYC 505 by varied collection centers [***38, 39***]. A genetically engineered industrial yeast strain NRRL Y-50463 from ARS Patent Culture Collection was the subject of the investigation. Strain Y-50463 was a genetically modified strain from a fermentation inhibitor-tolerant variant of strain Y-12632. It contains a synthesized yeast codon optimized xylose isomerase gene *YXI* [***32, 33***] in its chromosome XV and a set of heterologous xylose utilization genes carried by a plasmid, including xylitol dehydrogenase (*XYL2*), xylulokinase (*XKS1*), and two xylose transporter genes *XUT4* and *XUT6* from *S*. *stipitis* [***28, 33***]. The lyophilized cultures were recovered on YP medium and maintained on the YP medium supplemented with or without 25 g/L D-xylose.

### Culture conditions and sampling

Utilization and ethanol production of glucose and xylose was evaluated under aerobic and oxygen-limited fermentation conditions separately on YP medium containing 25 g/L D- glucose and 25 g/L D-xylose. For aerobic growth experiment, inoculum cells were prepared from YP medium containing xylose only. Cultures with an initial OD_600_ reading at 0.1 were incubated using a fleaker system [***30***] at 30°C with agitation at 250 rpm and cell growth was monitored by absorbance at OD_600._ For oxygen-limited fermentation experiments, a cell mass at 5 g/L was prepared on YP medium containing glucose only to save time building the cell mass required and to facilitate an immediate fermentation process. Tubes on fleaker covers were sealed to maintain oxygen-limited conditions for the fermentation. Two replicated experiments were carried out for each of the aerobic and oxygen-limited test set separately. Cell samples were taken periodically, frozen on dry ice, and then stored at -20°C until use for total RNA extraction. Cell free supernatants from each time point were collected for metabolic profile analysis using a Shimadzu high-performance liquid chromatography (HPLC) as previously described [***40***].

### The qRT-PCR array

A set of 96-well qRT-PCR array was made containing 86 genes involved in glycolysis, pentose phosphate pathway, and tricarboxylic acid (TCA) cycle. To ensure reproducibility and comparability of qRT-PCR data, a standard mRNA quality control reference was applied for the multiple-plate qRT-PCR array assay. Five external mRNA species, beta-2-microglobulin (*B2M*), major latex protein (*MSG*), chlorophyll A-B binding protein of LHCI type III precursor (*CAB*), ribulose bisphosphate carboxylase small chain 1 precursor (*RBS1*), and beta-actin (*ACTB*), were synthesized *in vitro* following procedures described previously [***41***]. The external mRNA reference was prepared in a mix consisting of accurately calibrated mRNA transcripts of *MSG*, *CAB*, *RBS1*, and *ACTB* at 0.1, 1, 10, and 1000 pg per μl, respectively. A standard curve was constructed for each qRT-PCR run using the mRNA reference as a calibration standard. Reactions for the reference genes were placed on the top of each 96-well plate with two replications. Reactions of the 86 target genes were arranged in the remaining wells on the plate. Two plates of replicated reactions were made for each sample serving as technical replications for each condition from strain Y-12632 and Y-50463 separately. Two biological replications were carried out for all samples at each time-point.

Genes involved in pathways of glycolysis, pentose phosphate pathway and TCA cycle were selected based on the KEGG database [***42***]. Primers for these genes and five heterologous xylose-utilizing genes *YXI*, *XYL2*, *XKS1*, *XUT4* and *XUT6* were designed based on the *YXI* sequence [***32***] and *Scheffersomyces stipitis* genome sequence [***42, 43***] with an aid of primer screening procedure using Primer 3 software. Primers used in this study are presented (S1 Table) with designed amplicon length ranging from 100 to 150 bp for target genes.

### Conditions and profiles of qRT-PCR

Total RNA was isolated and RNA integrity was verified by gel electrophoresis and NanoDrop Spectrophotometer ND-100 (NanoDrop Technologies, Inc., Wilmington, DE) as previously described [***31, 37, 44***]. Reverse transcription reaction was prepared by adding 1 μl of external mRNA reference consisting of a set of the above mentioned accurately calibrated mRNA transcripts into 2 μg of a host total RNA, 0.5 μg of oligo (dT)_18_, and 1 μ of 10 mM of dNTP mix. The volume was adjusted by water to 13 μl, then mixed well and incubated at 65°C for 5 min. The reaction tubes were chilled on ice for at least 1 min. Then added 4 μl 5X first strand buffer, 1 μl of 0.1M DTT, 1 μl SuperScript III (200 U/μl) (Invitrogen, CA), and 1 μl RNaseOUT (40 U/μl) (Invitrogen, CA) with a final volume of 20 μl. The reaction was incubated at 50°C for 1 h, 70°C for 15 min, and 4°C to end the reaction using a PCR cycler. SYBR Green iTaq PCR master mix (BioRad Laboratories) was applied for each qRT-PCR prep. For each reaction, a total of 25 μl was used consisting of 12.5 μl 2X SYBR Green MasterMix, 0.5 μl each of forward and reverse primer (10 μM each), 0.25 μl cDNA template and 11.25 μl H_2_O. PCR was run on an ABI Sequence Detection 7500 System using the following thermal profile: stage1: 95°C for 3 min; stage 2: 40 cycles of 95°C for 15 sec and 60°C for 45 sec; stage 3: 95°C for 15 sec, 60°C for 1 min and 95°C for 15 sec; stage 4: run dissociation curve with 95°C for 15 sec, 60°C for 1 min and 95°C for 15 sec. Stat Collection was set at stage 2 step 2 (60°C for 45 sec). A PCR reaction with a pair of primers for *B2M* and without a *B2M* template was used to serve as a negative control. A laboratory protocol entitled “Quantitative real-time RT-PCR assay applying Calibrated mRNA reference (Ctrl Mix)” is available in protocols.io with the following DOI: http://dx.doi.org/10.17504/protocols.io.nradd2e.

### Data analysis

To guard a reproducible and comparable data analysis, the build-in *Auto* data acquisition option with the instrument was quitted. Instead, a *Manual* option was applied in qRT-PCR data acquisition for each PCR run. Mean value of the *CAB* (with 1 pg spiked-in mRNA in the quality control mix) amplifications on each plate was designated as a sole reference to set up a *Manual* cycle threshold (Ct) at 26 for each plate as previously described [***36, 44***]. Data were analyzed after normalization by this mRNA reference. A standard curve was generated for each qRT-PCR plate. Upon completion of all reactions, a master equation was established and used for quantitative data analysis following previously described procedures [***37***]. The mRNA mass for each gene was obtained by an anti-log conversion. For absolute quantification of gene expression analysis, transcription number for each target genes was calculated using an equation as previously described [***37, 45***]. For each strain of Y-12632 and Y-50463, a transcription copy number at various time-points was normalized with its own at the 0 h for aerobic growth and oxygen-limited fermentation conditions separately. The differential expression at each time-point was presented by fold changes of Y-50463 over Y-12632 using the above normalized values in the comparative analysis.

## Results

### Cell growth and sugar consumption under aerobic conditions

**Fig 2:**
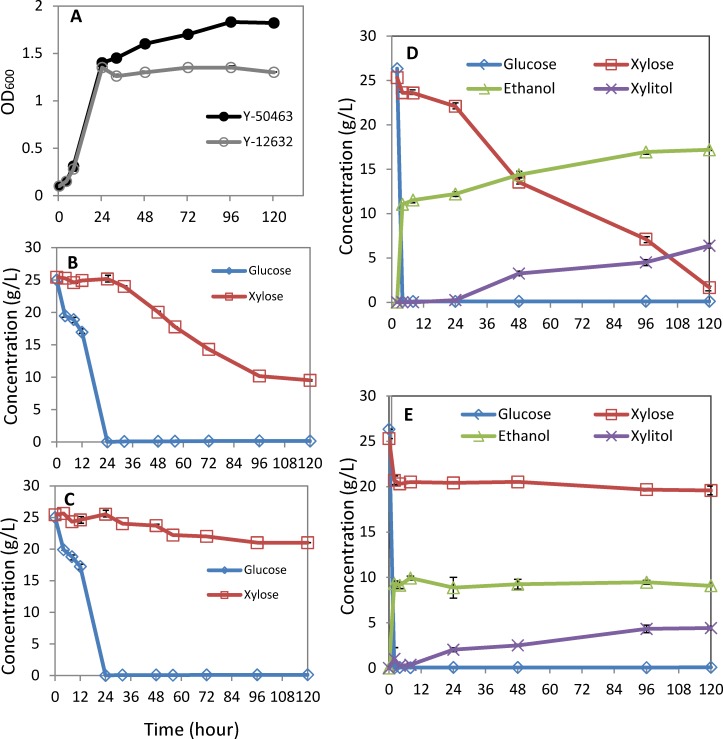
Comparison of strain response. Comparison of cell growth (A) and sugar consumption of genetically engineered *Saccharomyces cerevisiae* NRRL Y-50463 (B) and its parental wild type industrial yeast strain NRRL Y-12632 (C) on a medium containing mixed sugars of glucose and xylose each at 25g/L under aerobic conditions; and ethanol production for Y-50463 (D) and Y-12632 (E) under oxygen-limited fermentation conditions.

Under aerobic conditions on YP medium containing mixed sugars of glucose and xylose each at 25g/L, both wild-type strain Y-12632 and the engineered strain Y-50463 showed a rapid growth and reached to 1.4 OD reading at 24 h after incubation (Fig 2A). Then cell density of strain Y-50463 was increased continuously reaching the highest OD of 1.85 at 96 h. In contrast, there was no increased cell growth was observed for the wild-type Y-12632 until 120 h (Fig 2A). Both strains showed a similar pattern of glucose consumption which was exhausted at 24 h as indicated by HPLC analysis (Fig 2B and 2C). Strain Y-50463 consumed a large portion of xylose, but strain Y-12632 showed no significant xylose consumption and most xylose was recovered remaining in the medium until 120 h. Under the aerobic growth conditions, there was no significant ethanol production was observed.

### Ethanol production

Under oxygen-limited fermentation conditions, strain Y-50463 quickly exhausted glucose and consumed xylose in a nearly linear pattern toward the end of the fermentation (Fig 2D). Since a higher amount of cell mass at 5g/L was introduced to accelerate an immediate fermentation process, no further cell mess was measured under the oxygen-limited fermentation conditions. The highest concentration of ethanol production of 17 g/L was observed at 48 h. It produced approximately 6 g/L xylitol. Its parental strain Y-12632 showed similar trend of glucose consumption but no utilization of xylose throughout the fermentation by HPLC assay (Fig 2E). The highest concentration of its ethanol production only reached to 9.4 g/L which was mainly from glucose. The xylitol conversion was recovered at approximately 4 g/L at the end of the fermentation.

### Quantitative analysis of gene expression

**Fig 3:**
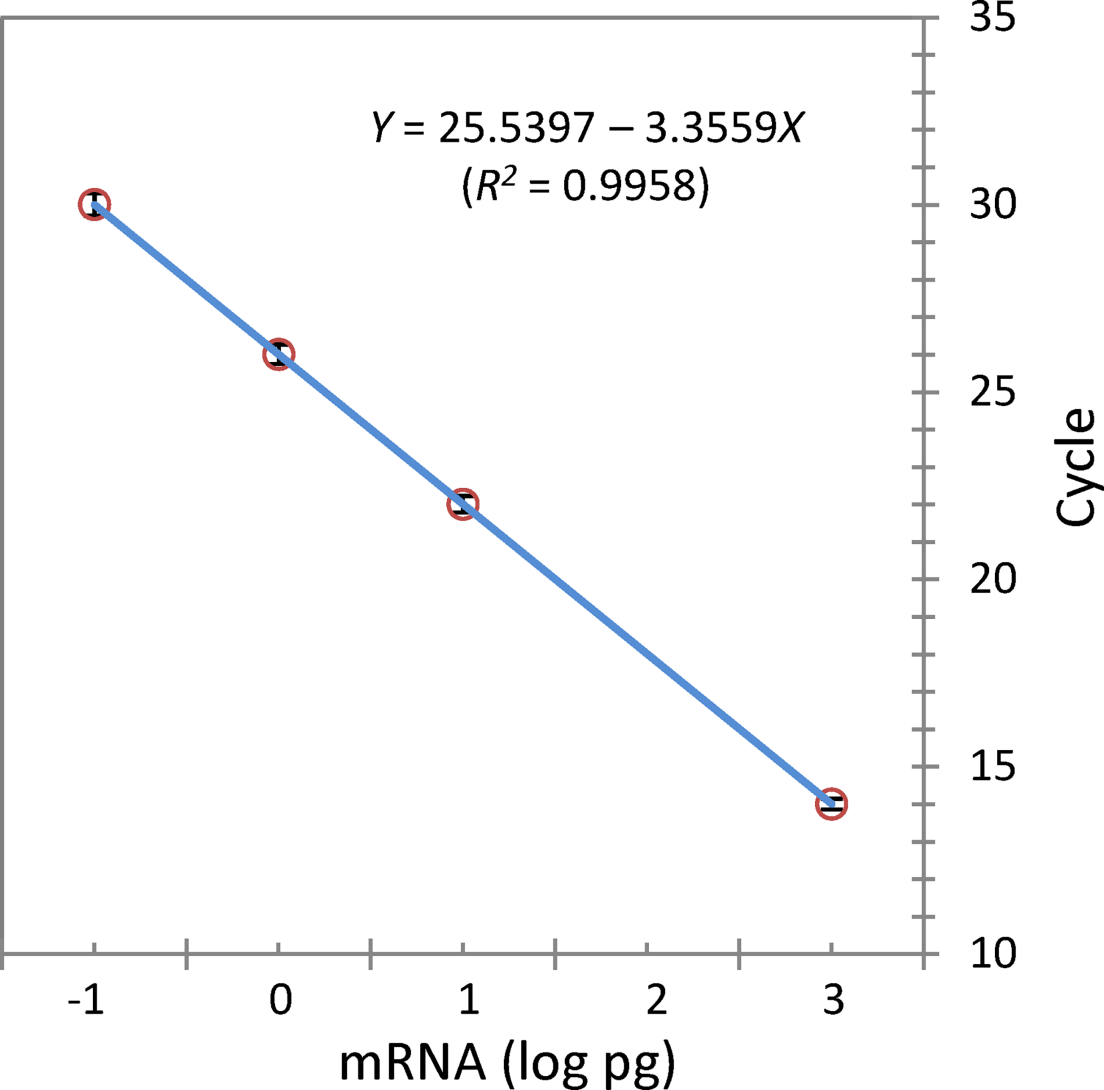
Master equation. A master equation generated based on all qRT-PCR reactions in this study using the universal RNA reference performed on the ABI Sequence Detection 7500 System. The slope of -3.3559 indicated an amplification efficiency of 0.986 for the qRT-PCR reactions in this study.

Application of the universal external RNA reference safe guarded the reproducibility and comparability of data obtained from the qRT-PCR array assays. Based on all qRT-PCR reactions, a master equation was established (Fig 3) as follows:

$$Y=25.5397–3.3559X\left(R^{2}=0.9958\right)$$ (1)

where variable *Y* stands for the cycle value of the qRT-PCR; variable *X* represents the quantified mass of mRNA (log pg); and constant 25.5397 or -3.3559 represents a constant Ct or slope, respectively, for the qRT-PCR in this study. The slope is an important quality control measurement indicating the amplification efficiency of the qRT-PCR. The slope of -3.3559 for the highly fitted linear relationship obtained in this investigation represented a high quality of qRT-PCR performance with an average amplification efficiency of 98.6% [***37, 46***]. Following an anti-log conversion, a transcription number for each target gene was obtained for comparative gene expression analysis between the two strains.

### Heterologous gene expression

**Fig 4:**
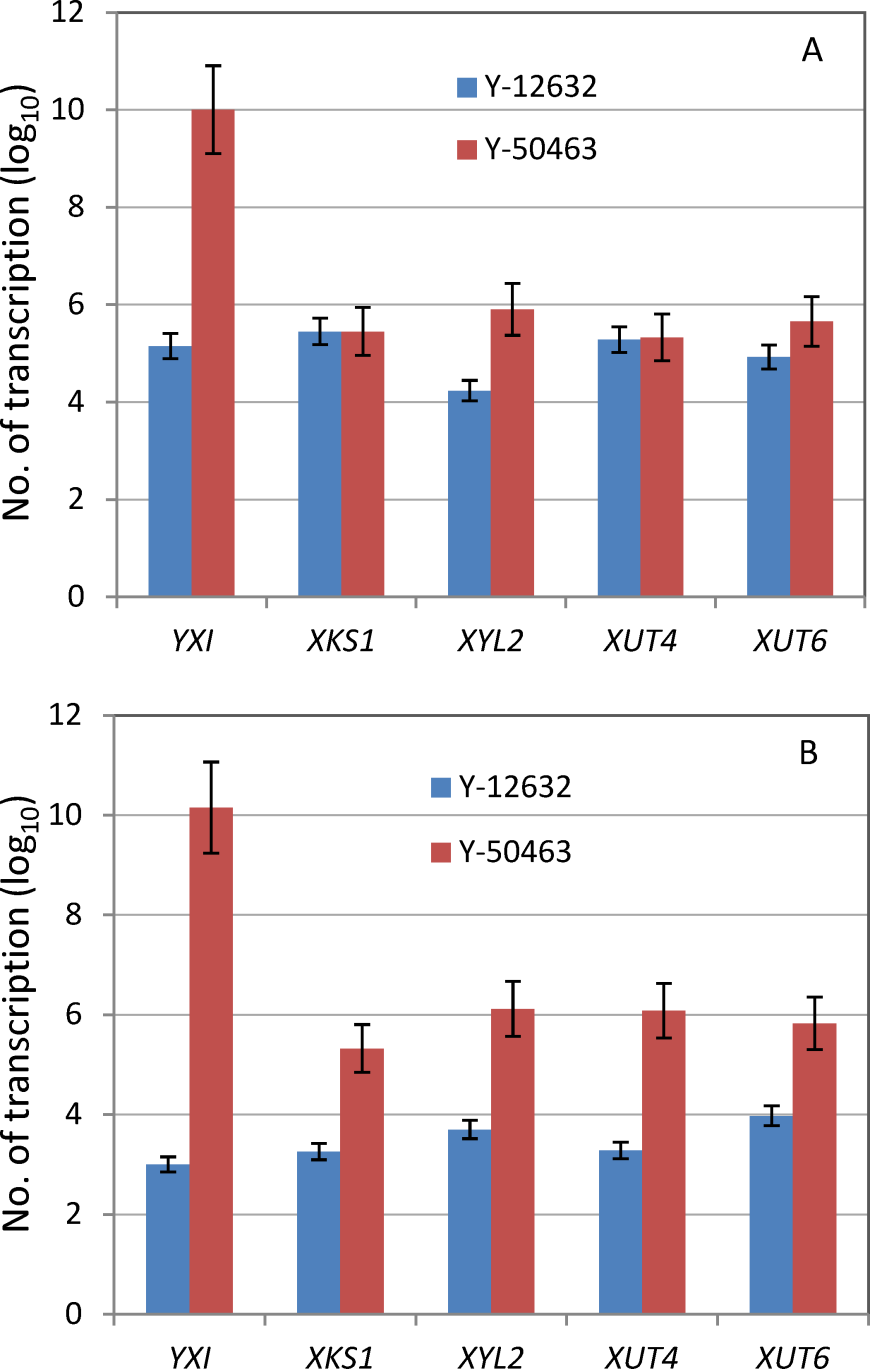
Hetrologous gene expression. Quantitative expression of five heterologous genes in the genetically engineered *Saccharomyces cerevisiae* NRRL Y-50463 in comparison with its parental wild type industrial yeast strain NRRL Y-12632 by qRT-PCR analysis at 4 h (A) and 24 h (B) after incubation under aerobic growth conditions.

The yeast codon optimized *YXI* genetically integrated into the chromosome XV of strain Y-50463 displayed an extremely higher level of expression with 1.03 x 10^10^ transcriptions at 4 h after incubation under aerobic growth conditions, which was 7000-fold increase than the wild type control (Fig 4A). Xylitol dehydrogenase gene (*XYL2*) and xylose transporter gene *XUT6* from *S*. *stipitis* carried in a plasmid showed a transcription level of 7.9 x 10^5^ and 4.5 x 10^5^ representing 45- and 5-fold increase than the control, respectively. However, no significant expression was observed for xylulokinase gene (*XKS1*) and xylose transporter gene *XUT4* compared with the control strain Y-12632 at 4 h after incubation (Fig 4A). Expression levels of all these genes were increased significantly 24 h after incubation. *YXI* maintained the highest level of enhanced expression reaching to 1.4 x 10^10^ transcriptions, more than 13,000-fold increase compared with the control (Fig 4B). *XYL2* and XKS1 increased 250- and 100-fold, respectively. Xylose transporter genes *XUT4* displayed more than 600-fold increase and *XUT6* only increased 70-fold. A similar trend of expression was observed under the oxygen-limited fermentation conditions (data not shown).

### Early gene expression response to the mixed sugars of glucose-xylose

Comparative gene expression analysis was conducted under both aerobic and oxygen-limited conditions. Cells grown on the medium containing both glucose (20 g/L) and xylose (25 g/L) at 4 h after incubation were used for aerobic growth treatment. In a total of 86 genes tested, 27 genes from strain Y-50463 showed significantly higher levels of expression compared with the parental strain Y-12632. Among which, 16 genes were identified to be involved in glycolysis, 5 in pentose phosphate pathway, and 6 in TCA cycle (Table 1). Several genes displayed extremely higher levels of up-regulated expressions ranged from 5- to 20-fold increases, such as *PRS4*, *HXK2*, *PCK1*, *FBP1*, and *ADH7*, compared with the parental control strain.

**Table 1:** Relative gene expression changes in ratio for *Saccharomyces cerevisiae* NRRL Y-50463 in comparison to its parental strain Y-12632 on a medium containing glucose and xylose under aerobic growth conditions.

Gene and category	Function description	Ratio		
		4h	24h	72h
*Glycolysis / Gluconrogenesis*				
*ACS1*	Acetyl-coA synthetase isoform	0.78	4.66*	0.09
*ACS2*	Acetyl-coA synthetase isoform	**2.03**	**1.59**	1.14
*ADH3*	Mitochondrial alcohol dehydrogenase isozyme III	0.69	**2.06**	1.13
*ADH4*	Alcohol dehydrogenase isoenzyme IV	0.35	0.49	0.11
*ADH5*	Alcohol dehydrogenase isoenzyme V	**2.63**	**1.65**	0.12
*ADH7*	NADPH-depend entalcohol dehydrogenase	**20.74**	**17.08**	0.62
*ALD2*	Cytoplasmic aldehyde dehydrogenase; uses NAD+ as the preferred coenzyme	-	**6.82**	0.02
*ALD3*	Cytoplasmic aldehyde dehydrogenase; uses NAD+ as the preferred coenzyme	0.29	**4.21**	**1.84**
*ALD4*	Mitochondrial aldehyde dehydrogenase; utilizes NADP+ or NAD+ equally as coenzymes	1.03	0.89	0.4
*ALD5*	Mitochondrial aldehyde dehydrogenase; utilizes NADP+ as the preferred coenzyme	**2**	**9.23**	**2.18**
*ALD6*	Cytoplasmic aldehyde dehydrogenase; uses NADP+ as the preferred coenzyme	**1.74**	1.09	0.1
*CDC19*	Pyruvate kinase	**2.96**	**1.65**	0.61
*ENO1*	Enolase I	0.51	0.94	0.2
*ENO2*	Enolase II	0.32	0.42	0.06
*FBA1*	Fructose 1,6-bisphosphate aldolase	0.87	**2.27**	0.11
*FBP1*	Fructose-1,6-bisphosphatase, key regulatory enzyme in the gluconeogenesis pathway	**12.4**	**5.77**	-
*GLK1*	Glucokinase	0.86	0.86	0.53
*GPM1*	Tetrameric phosphoglycerate mutase	0.59	0.56	0.53
*GPM2*	Homolog of Gpm1p phosphoglycerate mutase	**2.96**	**5.59**	0.06
*GPM3*	Homolog of Gpm1p phosphoglycerate mutase	**2.89**	**5.99**	**9.04**
*HXK1*	Hexokinase isoenzyme 1	0.66	0.89	0.59
*HXK2*	Hexokinase isoenzyme 2; functions in the nucleus to repress expression of HXK1 and GLK1	**7.02**	**6.9**	0.01
*PCK1*	Phosphoenolpyruvate carboxykinase, key enzyme in gluconeogenesis	**7.23**	**1.79**	1.68
*PDA1*	E1 alpha subunit of the pyruvate dehydrogenase (PDH) complex	0.98	0.74	0.08
*PDB1*	E1 beta subunit of the pyruvate dehydrogenase (PDH) complex	0.52	0.84	0.01
*PDC1*	Major of three pyruvate decarboxylase isozymes	0.51	1.17	0.59
*PDC5*	Minor isoform of pyruvate decarboxylase	**1.93**	**1.74**	0.91
*PDC6*	Minor isoform of pyruvate decarboxylase	0.79	0.95	0.03
*PFK1*	Alpha subunit of heterooctameric phosphofructokinase	1.18	**2.3**	**2.47**
*PFK2*	Beta subunit of heterooctameric phosphofructokinase	1.26	**3.16**	1.38
*PGI1*	Glycolytic enzyme phosphoglucose isomerase	**2.47**	**2.36**	0.58
*PGK1*	3-phosphoglycerate kinase	0.36	0.94	0.76
*PGM1*	Phosphoglucomutase, minor isoform	**2.63**	**3.12**	1.28
*PGM2*	Phosphoglucomutase	**1.88**	**1.68**	-
*PGM3*	Phosphoglucomutase	0.88	1.35	0.24
*PYK2*	Pyruvate kinase	**1.53**	**3.12**	0.16
*SFA1*	Bifunctional alcohol dehydrogenase and formaldehyde dehydrogenase	1.31	**2.84**	**1.86**
*TDH1*	Glyceraldehyde-3-phosphate dehydrogenase, isozyme 1	0.53	1.15	0.97
*TDH2*	Glyceraldehyde-3-phosphate dehydrogenase, isozyme 2	0.48	0.73	0.67
*TDH3*	Glyceraldehyde-3-phosphate dehydrogenase, isozyme 3	0.39	0.66	0.66
*THI3*	Probable alpha-ketoisocaproate decarboxylase	0.76	**2.24**	0.51
*TPI1*	Triose phosphate isomerase	0.49	1.09	0.59
*Pentose phosphate pathway*				
*GND1*	6-phosphogluconate dehydrogenase; NADPH regenerating reaction	0.13	0.21	0.01
*GND2*	6-phosphogluconate dehydrogenase; NADPH regenerating reaction	0.53	0.01	0.01
*NQM1*	Transaldolase of unknown function	0.7	**2.71**	0.19
*PRS1*	5-phospho-ribosyl-1(alpha)-pyrophosphate synthetase	0.62	**1.61**	1.31
*PRS2*	5-phospho-ribosyl-1(alpha)-pyrophosphate synthetase	**2.25**	**6.14**	0.27
*PRS3*	5-phospho-ribosyl-1(alpha)-pyrophosphate synthetase	1.17	1.21	1.39
*PRS4*	5-phospho-ribosyl-1(alpha)-pyrophosphate synthetase	**5.98**	**5.13**	0
*PRS5*	5-phospho-ribosyl-1(alpha)-pyrophosphate synthetase	**2.6**	**4.26**	**4.95**
*RBK1*	Putative ribokinase	0.88	**1.65**	0.31
*RKI1*	Ribose-5-phosphate ketol-isomerase	0.82	1.38	**1.73**
*RPE1*	D-ribulose-5-phosphate 3-epimerase	1.13	0.61	0.01
*SOL1*	Protein with a possible role in tRNA export	**2.21**	**3.49**	**1.85**
*SOL2*	Protein with a possible role in tRNA export	0.6	0.86	0.25
*SOL3*	6-phosphogluconolactonase	0.26	1	0.34
*SOL4*	6-phosphogluconolactonase	**2.5**	0.94	0.05
*TAL1*	Transaldolase	0.43	**1.73**	0.6
*TKL1*	Transketolase	0.67	**2.16**	0.5
*TKL2*	Transketolase	1.21	**1.54**	1.46
*ZWF1*	Glucose-6-phosphate dehydrogenase (G6PD)	0.8	0.58	0.36
*TCA cycle*				
*ACO1*	Aconitase	0.93	1.34	0.69
*ACO2*	Putative mitochondrial aconitase isozyme	1.08	**1.71**	0.86
*CIT1*	Citrate synthase	1.2	**2.3**	1.07
*CIT2*	Citrate synthase	**1.98**	**4.5**	**6.28**
*CIT3*	Dual specificity mitochondrial citrate and methylcitrate synthase	**1.8**	**2.82**	1
*FUM1*	Fumarase	1.03	1.43	0.78
*IDH1*	Subunit of mitochondrial NAD(+)-dependent isocitrate dehydrogenase	0.34	**1.53**	**1.56**
*IDH2*	Subunit of mitochondrial NAD(+)-dependent isocitrate dehydrogenase	0.74	**1.55**	0.56
*IDP1*	Mitochondrial NADP-specific isocitrate dehydrogenase	0.49	**2.8**	**1.95**
*IDP2*	Cytosolic NADP-specific isocitrate dehydrogenase	1.21	1.38	0.58
*IDP3*	Peroxisomal NADP-dependent isocitrate dehydrogenase	1.36	**1.91**	0.75
*KGD1*	Component of the mitochondrial alpha-ketoglutarate dehydrogenase complex	0.83	**2.25**	1.06
*KGD2*	Dihydrolipoyl transsuccinylase	0.92	**2.94**	**1.83**
*LSC1*	Alpha subunit of succinyl-CoA ligase	0.76	0.47	0.22
*LSC2*	Beta subunit of succinyl-CoA ligase	1.12	**1.7**	0.64
*MDH1*	Mitochondrial malate dehydrogenase	**1.71**	1.32	0.73
*MDH2*	Cytoplasmic malate dehydrogenase	**2.21**	**3.1**	0.49
*MDH3*	Peroxisomal malate dehydrogenase	1.16	0.75	0.19
*PYC1*	Pyruvate carboxylase isoform	**2.59**	**5.24**	0.6
*PYC2*	Pyruvate carboxylase isoform	0.66	**1.87**	0.8
*SDH1*	Flavoprotein subunit of succinate dehydrogenase	0.88	**1.62**	0.53
*SDH2*	Iron-sulfur protein subunit of succinate dehydrogenase	0.58	**1.72**	0.66
*SDH3*	Subunit of both succinate dehydrogenase and of TIM22 translocase	0.44	0.19	0.15
*SDH4*	Membrane anchor subunit of succinate dehydrogenase	0.77	-	0.34
*YJL045W*	Minor succinate dehydrogenase isozyme	**1.6**	**1.87**	0.16

* Numbers in bold indicate a significant differential expression ratio above 1.5.

Since the glucose was depleted completely in the medium 4 h after fermentation under oxygen-limited conditions, cell samples were taken at 2 h and used for gene expression analysis in response to the mixed sugars. During the early hours of ethanol production in the presence of glucose and xylose, most genes maintained normal or nearly normal levels of expression. Under the oxygen-limited conditions, only six genes out of 42 from glycolysis and two genes out of 19 from pentose phosphate pathway showed increased expressions (Table 2). Under oxygen-limited conditions, a small number of genes showed similar repressed expressions such as *ADH4*, *ENO2*, *GND1* and *GND2*. These genes were also repressed under aerobic growth conditions. Several other genes shared increased expressions under both conditions such as *ALD5*, *GPM2*, *GPM3*, *HXK2*, *PRS4* and *PRS5* which are involved in glycolysis and pentose phosphate pathways.

**Table 2:** Relative gene expression changes in ratio for *Saccharomyces cerevisiae* NRRL Y-50463 in comparison to its parental strain Y-12632 on a medium containing glucose and xylose under oxygen-limited fermentation conditions.

Gene and category	Function description	Ratio		
		2h	24h	48h
*Glycolysis / Gluconrogenesis*				
*ACS1*	Acetyl-coA synthetase isoform	1.53*	0.41	0.88
*ACS2*	Acetyl-coA synthetase isoform	0.31	0.91	1.63
*ADH2*	Alcohol dehydrogenase isoenzyme II	1.03	**1.81**	1.06
*ADH3*	Mitochondrial alcohol dehydrogenase isozyme III	1.26	**2.24**	1.45
*ADH4*	Alcohol dehydrogenase isoenzyme IV	0.1	0.45	0.88
*ADH5*	Alcohol dehydrogenase isoenzyme V	0.09	0.77	1.6
*ADH7*	NADPH-depend entalcohol dehydrogenase	0.99	0.49	0.93
*ALD2*	Cytoplasmic aldehyde dehydrogenase; uses NAD+ as the preferred coenzyme	**1.72**	**3.15**	**4.04**
*ALD3*	Cytoplasmic aldehyde dehydrogenase; uses NAD+ as the preferred coenzyme	1.13	1.07	1.13
*ALD4*	Mitochondrial aldehyde dehydrogenase; utilizes NADP+ or NAD+ equally as coenzymes	1.21	1.23	1.24
*ALD5*	Mitochondrial aldehyde dehydrogenase; utilizes NADP+ as the preferred coenzyme	**2.07**	1.22	1.06
*ALD6*	Cytoplasmic aldehyde dehydrogenase; uses NADP+ as the preferred coenzyme	1.35	0.62	1.42
*CDC19*	Pyruvate kinase	0.59	0.88	1.55
*ENO1*	Enolase I	0.92	1.02	1.25
*ENO2*	Enolase II	0.42	0.33	0.78
*FBA1*	Fructose 1,6-bisphosphate aldolase	1.07	**3.55**	1.32
*FBP1*	Fructose-1,6-bisphosphatase, key regulatory enzyme in the gluconeogenesis pathway	1.19	0.88	0.69
*GLK1*	Glucokinase	1.4	1.14	1.21
*GPM1*	Tetrameric phosphoglycerate mutase	0.85	0.57	1.16
*GPM2*	Homolog of Gpm1p phosphoglycerate mutase	**2.11**	0.75	0.97
*GPM3*	Homolog of Gpm1p phosphoglycerate mutase	**3.32**	**2.61**	1
*HXK1*	Hexokinase isoenzyme 1	0.77	1.38	1.29
*HXK2*	Hexokinase isoenzyme 2; functions in the nucleus to repress expression of HXK1 and GLK1	**1.91**	**3.25**	**1.92**
*PCK1*	Phosphoenolpyruvate carboxykinase, key enzyme in gluconeogenesis	0.88	0.82	0.94
*PDA1*	E1 alpha subunit of the pyruvate dehydrogenase (PDH) complex	1.2	0.64	1.25
*PDB1*	E1 beta subunit of the pyruvate dehydrogenase (PDH) complex	1.05	0.67	1.03
*PDC1*	Major of three pyruvate decarboxylase isozymes	0.55	0.56	1.14
*PDC5*	Minor isoform of pyruvate decarboxylase	0.51	1.03	**1.53**
*PDC6*	Minor isoform of pyruvate decarboxylase	0.9	0.84	1.17
*PFK1*	Alpha subunit of heterooctameric phosphofructokinase	0.98	**3.63**	0.61
*PFK2*	Beta subunit of heterooctameric phosphofructokinase	1	**1.54**	1.24
*PGI1*	Glycolytic enzyme phosphoglucose isomerase	0.77	**4.68**	**2.36**
*PGK1*	3-phosphoglycerate kinase	0.77	1.18	1.32
*PGM1*	Phosphoglucomutase, minor isoform	0.93	0.83	1.13
*PGM2*	Phosphoglucomutase	0.67	**4.65**	1.01
*PGM3*	Phosphoglucomutase	0.77	1.05	1.26
*PYK2*	Pyruvate kinase	0.89	0.94	0.84
*SFA1*	Bifunctional alcohol dehydrogenase and formaldehyde dehydrogenase	0.88	**2.11**	**1.84**
*TDH1*	Glyceraldehyde-3-phosphate dehydrogenase, isozyme 1	0.6	0.97	**1.55**
*TDH2*	Glyceraldehyde-3-phosphate dehydrogenase, isozyme 2	1.06	0.5	**2.54**
*TDH3*	Glyceraldehyde-3-phosphate dehydrogenase, isozyme 3	0.9	0.47	0.52
*THI3*	Probable alpha-ketoisocaproate decarboxylase	1.4	0.7	0.99
*TPI1*	Triose phosphate isomerase	0.85	**2.11**	**1.57**
*Pentose phosphate pathway*				
*GND1*	6-phosphogluconate dehydrogenase; NADPH regenerating reaction	0.68	0.75	0.71
*GND2*	6-phosphogluconate dehydrogenase; NADPH regenerating reaction	0.43	1.31	**1.52**
*NQM1*	Transaldolase of unknown function	0.73	**3.28**	1.39
*PRS1*	5-phospho-ribosyl-1(alpha)-pyrophosphate synthetase	0.92	**1.56**	1.21
*PRS2*	5-phospho-ribosyl-1(alpha)-pyrophosphate synthetase	1.03	**3.78**	1.27
*PRS3*	5-phospho-ribosyl-1(alpha)-pyrophosphate synthetase	0.64	0.71	0.94
*PRS4*	5-phospho-ribosyl-1(alpha)-pyrophosphate synthetase	**2.05**	**2.44**	**4.59**
*PRS5*	5-phospho-ribosyl-1(alpha)-pyrophosphate synthetase	**3.26**	**3.88**	**1.58**
*RBK1*	Putative ribokinase	0.7	0.29	0.9
*RKI1*	Ribose-5-phosphate ketol-isomerase	1.17	0.99	1.16
*RPE1*	D-ribulose-5-phosphate 3-epimerase	1.08	1.16	**1.59**
*SOL1*	Protein with a possible role in tRNA export	1.34	**2.14**	1.25
*SOL2*	Protein with a possible role in tRNA export	0.73	1	0.96
*SOL3*	6-phosphogluconolactonase	1.09	0.53	0.51
*SOL4*	6-phosphogluconolactonase	0.46	**1.54**	0.79
*TAL1*	Transaldolase	0.89	**2.68**	1.01
*TKL1*	Transketolase	0.91	**1.65**	1.25
*TKL2*	Transketolase	1.03	**1.63**	1.31
*ZWF1*	Glucose-6-phosphate dehydrogenase (G6PD)	0.62	1.01	1.19

* Numbers in bold indicate a significant differential expression ratio above 1.5.

### Gene expression response to xylose after depletion of glucose

**Fig 5:**
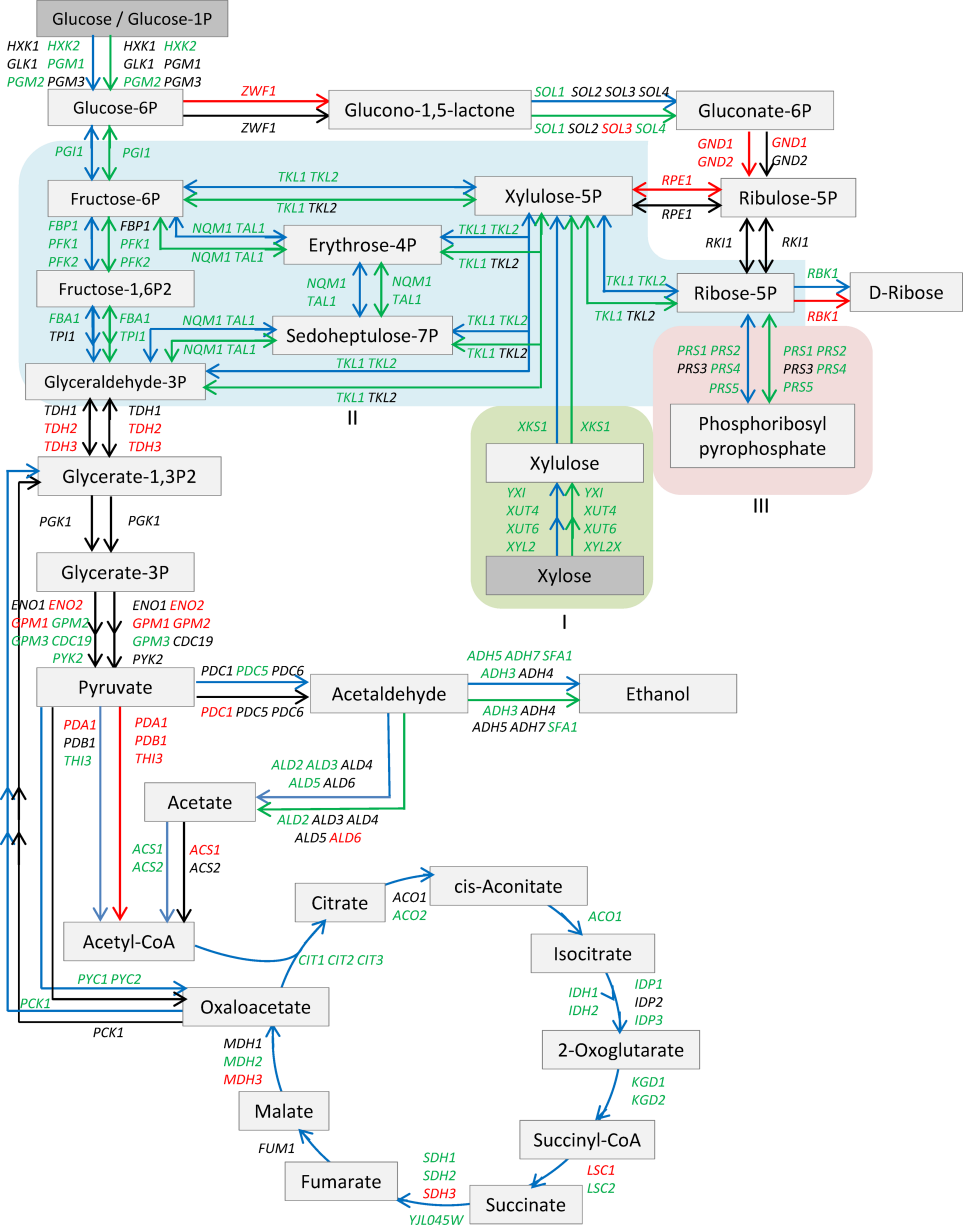
Signature expression pathway. A schematic illustration of significant gene expression changes for the genetically engineered *Saccharomyces cerevisiae* NRRL Y-50463 compared with its parental wild type industrial yeast strain NRRL Y-12632 for endogenous genes involved in glycolysis, pentose phosphate pathway and TCA cycle at 24 h using xylose as the sole source of carbon when glucose was depleted. Arrows on the left and the top from the parallel lines represent aerobic growth condition and those on the right side or at the bottom represent oxygen-limited fermentation condition. Blue or green colored arrows indicate significantly greater gene expression for aerobic and oxygen-limited condition, respectively. Arrows in red indicate repressed expression and arrows in black indicate gene expression at normal or nearly normal levels. Elements of the signature expression for strain NRRL Y-50463 were boxed in varied colors and marked as I, II and III, respectively.

Glucose content in the medium was completely depleted for both strains Y-50563 and Y-12632 24 h after incubation under both aerobic and oxygen-limited conditions. At this stage, xylose was the only source of carbon for cell utilization. Under aerobic growth conditions, most genes from the genetically engineered strain Y-50463 showed increased expressions in response to xylose utilization compared with its parental strain Y-12632. There were 24 genes out of 42 genes tested in glycolysis, 10 out of 19 tested in pentose phosphate pathway, and 17 out of 25 tested in TCA cycle, showed significantly higher levels of increased expression (Table 1 and Fig 5). Except for a few, many remaining genes showed normal or nearly normal levels of expression. A similar trend of expression patterns was observed at 24 h under oxygen-limited fermentation conditions for strain Y-50463. While most genes displayed normal or nearly normal expressions for xylose utilization, only 12 genes out of 42 genes tested, and 10 out of 19 showed significantly higher levels of increased expressions for glycolysis and pentose phosphate pathways, respectively (Table 2 and Fig 5). However, many genes maintained a sound expression level at 24 h and others showed improved expressions with the continued xylose utilization at 48 h. No expression analysis was conducted for TCA cycle genes under the oxygen-limited fermentation conditions.

Unlike observed for the early response to the mixed sugars, many genes showing increased expressions were overlapped for both aerobic and oxygen-limited conditions when glucose was depleted and xylose left as the only source of carbon. All genes showing significantly increased expression under the oxygen-limited condition were also consistently displayed higher levels of expression under the aerobic conditions, except for *SOL4* with a robust normal expression, for strain Y-50463.

### Signature expression of NRRL Y-50463

Since strain Y-50463 received five heterologous genes of *YXI*, *XUT4*, *XUT6*, *XYL2* and *XKS1* in its chromosome or cytoplasm, the enriched genetic background and the expression of these heterologous genes were naturally unique for the genetically engineered strain in contrast to its wild-type parental strain Y-12632 (Figs 4 and 5). The high level of constitutive expression by *YXI* was outstanding. Even for the other four genes showing relatively lower levels of expression, they were distinctly presented in the yeast and changed the yeast performance. These *YXI*-led five xylose-utilization facilitating genes signified a system response to adjust the Y-50463 performance, which induced significantly altered interactive expression relationships of the endogenous genes of the yeast.

Another outstanding pattern of the signature expression was observed for 10 genes, *TAL1*, *NQM1*, *TKL1*, *TKL2*, *PGI*, *FBP1*, *PFK1*, *PFK2*, *FBA1* and *TPI1*, involved in the non-oxidative pentose phosphate pathway at 24 h under both aerobic and oxygen-limited conditions (Tables 1 and 2 and Fig 5). Among these, four genes *TAL1*, *NQM1*, *TKL1* and *TKL2* were especially active in the pentose phosphate shunt pathway. These genes play significantly important roles in cell metabolism with multiple functions involving in GO categories of cellular component, molecular function and biological process (Table 3). Such a signature expression was distinct in contrast to the early expression response under both aerobic and oxygen-limited conditions. The other six genes important in this pathway were highly active and closely interactive with the upper portion of the glycolysis.

**Table 3:** Gene Ontology (GO) categories and terms for significantly induced endogenous genes of genetically engineered industrial yeast *Saccharomyces cerevisiae* NRRL Y-50463 on xylose-containing medium at 24h during oxygen-limited fermentation conditions.

GO ID	GO term	Gene
*Cellular component*		
GO:0005737	cytoplasm	PRS4*, ***PGI1***, *SFA1*, ***TPI1***, ***PRS2***, *HXK1*, ***HXK2***, ***PFK1***, *SOL4*, *GND2*, ***FBA1***, ***PRS1***, ***TAL1***, *ADH3*, ***PGM2***, *ALD2*, ***PFK2***, *SOL1*, *GPM3*, ***PRS5***, ***TKL1***
GO:0005945	6-phosphofructokinase complex	***PFK1***, ***PFK2***
GO:0005622	intracellular	***PGI1***, ***PGM2***, ***PRS5***, ***PRS2***, ***PFK1***, ***FBA1***, ***NQM1***, ***HXK2***, *ALD2*, ***TAL1***, *GPM3*, ***PRS4***, *ADH3*, *SOL1*, ***TPI1***, *SFA1*, ***TKL1***, ***PRS1***, *SOL4*, ***PFK2***, *HXK1*, *GND2*
*Molecular function*		
GO:0016491	oxidoreductase activity	*SFA1*, *ADH3*, *GND2*
GO:0016778	diphosphotransferase activity	***PRS4***, ***PRS2***, ***PRS1***, ***PRS5***
GO:0019200	carbohydrate kinase activity	*HXK1*, ***HXK2***, ***PFK1***, ***PFK2***
GO:0016740	transferase activity	***PRS4***, ***PRS2***, *HXK1*, ***HXK2***, ***NQM1***, ***PFK1***, ***PRS1***, ***TAL1***, ***PFK2***, ***PRS5***, ***TKL1***
GO:0016744	transferase activity, transferring aldehyde or ketonic groups	***NQM1***, ***TAL1***, ***TKL1***
GO:0003872	6-phosphofructokinase activity	***PFK1***, ***PFK2***
GO:0004736	intramolecular oxidoreductase activity, interconverting aldoses and ketoses	***PGI1***, ***TPI1***
*Biological process*		
GO:0019318	hexose metabolic process	***PGI1***, ***TPI1***, *HXK1*, ***HXK2***, ***PFK1***, *SOL4*, *GND2*, ***FBA1***, ***TAL1***, ***PGM2***, ***PFK2***, ***TKL1***
GO:0006006	glucose metabolic process	***PGI1***, ***TPI1***, *HXK1*, ***HXK2***, ***PFK1***, *SOL4*, *GND2*, ***FBA1***, ***TAL1***, ***PFK2***, ***TKL1***
GO:0019362	pyridine nucleotide metabolic process	***PGI1***, *SOL4*, *GND2*, ***TAL1***, *ADH3*, ***TKL1***
GO:0046391	5-phosphoribose 1-diphosphate metabolic process	***PRS4***, ***PRS2***, ***PRS1***, ***PRS5***
GO:0006733	oxidoreduction coenzyme metabolic process	***PGI1***, *SOL4*, *GND2*, ***TAL1***, *ADH3*, ***TKL1***
GO:0006740	NADPH regeneration	***PGI1***, *SOL4*, *GND2*, ***TAL1***, ***TKL1***
GO:0006091	generation of precursor metabolites and energy	***PGI1***, ***TPI1***, *HXK1*, ***HXK2***, ***PFK1***, ***FBA1***, ***PGM2***, ***PFK2***, *ADH3*
GO:0006739	NADP metabolic process	***PGI1***, *SOL4*, *GND2*, ***TAL1***, ***TKL1***
GO:0006793	phosphorus metabolic process	***PRS4***, ***PGI1***, ***PRS2***, *SOL4*, *GND2*, ***PRS1***, ***TAL1***, *ADH3*, ***PGM2***, ***PRS5***, ***TKL1***
GO:0009117	nucleotide metabolic process	***PGI1***, *SOL4*, *GND2*, ***TAL1***, *ADH3*, ***PGM2***, ***TKL1***
GO:0006732	coenzyme metabolic process	***PGI1***, *SOL4*, *GND2*, ***TAL1***, *ADH3*, ***TKL1***
GO:0055114	oxidation-reduction process	***PGI1***, *SOL4*, *GND2*, ***TAL1***, *ADH3*, ***PGM2***, ***TKL1***
GO:0000955	amino acid catabolic process via Ehrlich pathway	*SFA1*, *ADH3*
GO:0006000	fructose metabolic process	*HXK1*, ***HXK2***
GO:0046390	ribose phosphate biosynthetic process	***PRS4***, ***PRS2***, ***PRS1***, ***PRS5***
GO:0044281	small molecule metabolic process	***PGI1***, *SFA1*, *SOL4*, *GND2*, ***TAL1***, *ADH3*, ***PGM2***, *ALD2*, ***TKL1***
GO:0006098	pentose-phosphate shunt	*GND2*, ***PGI1***, *SOL4*, ***TAL1***, ***TKL1***
GO:0006096	glycolysis	***PGI1***,***TPI1***, *HXK1*, ***HXK2***, ***PFK1***, ***FBA1***, ***PFK2***
GO:1901564	organonitrogen compound metabolic process	***PGI1***, *SFA1*, *SOL4*, *GND2*, ***TAL1***, *ADH3*, *ALD2*, ***TKL1***

*Genes involved in the signature expression are bolded.

The third significant pattern of the signature expression for the genetically engineered yeast strain was observed for the *PRS* gene family including *PRS5*, *PRS4*, *PRS2*, *PRS1* and *PRS3* which is directly connected to the pathway of phosphoribosyl pyrophosphate (PRPP) (Tables 1, 2 and 3). This signature expression existed for the yeast cells grown under both aerobic and oxygen-limited conditions regardless of the stage of the early response or at 24 h. However, the expression levels of those genes were even higher on xylose when glucose was completely depleted (Fig 5).

## Discussion

Using pathway-based qRT-PCR array analyses, we demonstrated significantly higher levels of constitutive expression of *YXI* and revealed the insight into the signature pathway expression of the xylose utilization for the genetically engineered industrial yeast *S*. *cerevisiae* NRRL Y-50463 in this study. We identified three distinct signature expression patterns underlying the Y-50463 performance for its enabled xylose utilization capability, involving the following three groups of genes: I. A set of five heterologous genes engineered into Y-50463 including *YXI*, *XUT4*, *XUT6*, *XYL2* and *XKS1* involved in xylose-to-xylulose-5-phosphate conversion (Fig 5). II. Ten genes in the non-oxidative pentose phosphate pathway branch, especially for *TKL1* and *TKL2* or *TAL1* and *NQM1* which encode for transketolase or transaldolase enzymes, respectively for the serial of sugar transformation to drive the metabolic flow into glycolysis. III. The entire *PRS* gene family consisting of *PRS1*, *PRS2*, *PRS3*, *PRS4* and *PRS5* which encode 5-phospho-ribosyl-1(alpha)-pyrophosphate synthetases for synthesis of PRPP, a central compound for biosynthesis superpathway of nucleotide and amino acids. Knowledge obtained from the industrial yeast aids continued efforts in development of the next-generation biocatalyst for efficient lignocellulose-to-advanced biofuels conversion.

In the first element of the signature expression, the yeast codon optimized *YXI* genetically integrated into the chromosome XV of Y-50463 displayed a significantly higher level of constitutive expression in this study. Historically, *XI*-expressing *S*. *cerevisiae* strains suffered a lower rate of xylose fermentation despite an improved yield of ethanol [***47***]. Since *XI* is often expressed under promoters of multi-copy plasmids, its expression tends to be unstable especially under continuous cultivations [***29***]. In this study, the chromosomally integrated *YXI* with a robust promoter of *ADH1*safe guarded the *YXI* expression in Y-50463 [***33***]. The chromosomal location of the target gene was suggested to impact its expression and regulation [***48***]. In the design of Y-50463 used in this study, *YXI* was resided at the *ADH1* locus in chromosome XV (***33***). The superb high levels of *YXI* expression observed in this study can be benefited from its specific robust location in the chromosome. Such a constitutive expression of the *YXI* served a necessary driving force for xylose metabolism in Y-50463. Since xylose uptake and flux are limited by the sugar transport, efficient xylose transporters are necessary to improve the rate of xylose metabolism [***24, 27, 28***]. Introduction of a single xylose transporter gene in combination with *YXI* into an industrial yeast strain has been demonstrated to improve xylose uptake, volumetric consumption and increased ethanol production significantly [***28***]. In this study, two xylose transporter genes *XUT4* and *XUT6* appeared to facilitate the xylose transport function although their expression level was not compatible with that of *YXI*. Since these xylose transporter genes were carried by a single plasmid, the lower levels of the expression in contrast to the constitutive expression of *YXI* is expected due to a potential low copy number. Although *XUT4* and *XUT6* facilitated xylose uptake and consumption in this study, they are not the most efficient xylose transporter genes. Other xylose transporter genes such as *RGT2*, *SUT4* and *XUT7* were found to be more efficient than *XUT4* and *XUT6* under the same conditions [***28***]. We suggest these more efficient xylose transporter genes to be included for improvement in future genetic engineering efforts. In order to reduce xylitol accumulations caused by endogenous xylose reductase activity, *XYL2* was introduced into Y-50463 for the strain design [***33***]. However, such an approach did not completely eliminate xylitol and a residue amount of xylitol was still observed in this study. The expression of *XKS1* in Y-50463 in this study was also relatively lower than expected. It is likely caused by the large insert and the low copy number of the plasmid. Kinase reaction from xylulose into xylulose-5-phosphate is a key step to supply the basic intermediate into the non-oxidative pentose phosphate pathway branch. A constitutive expression of *XKS1* is needed for further improvement through combined efforts of sequence optimization and chromosomal integration. It is obvious that the introduction of a set the *YXI*-lead heterologous genes in Y-50463 changed gene expression profiles of the yeast. Consequently, the altered gene interactions activated xylose metabolism through the non-oxidative pentose phosphate pathway branch in Y-50463, which enabled xylose to be transformed into downstream of glycolysis for increased ethanol production (Fig 5).

**Fig 6:**
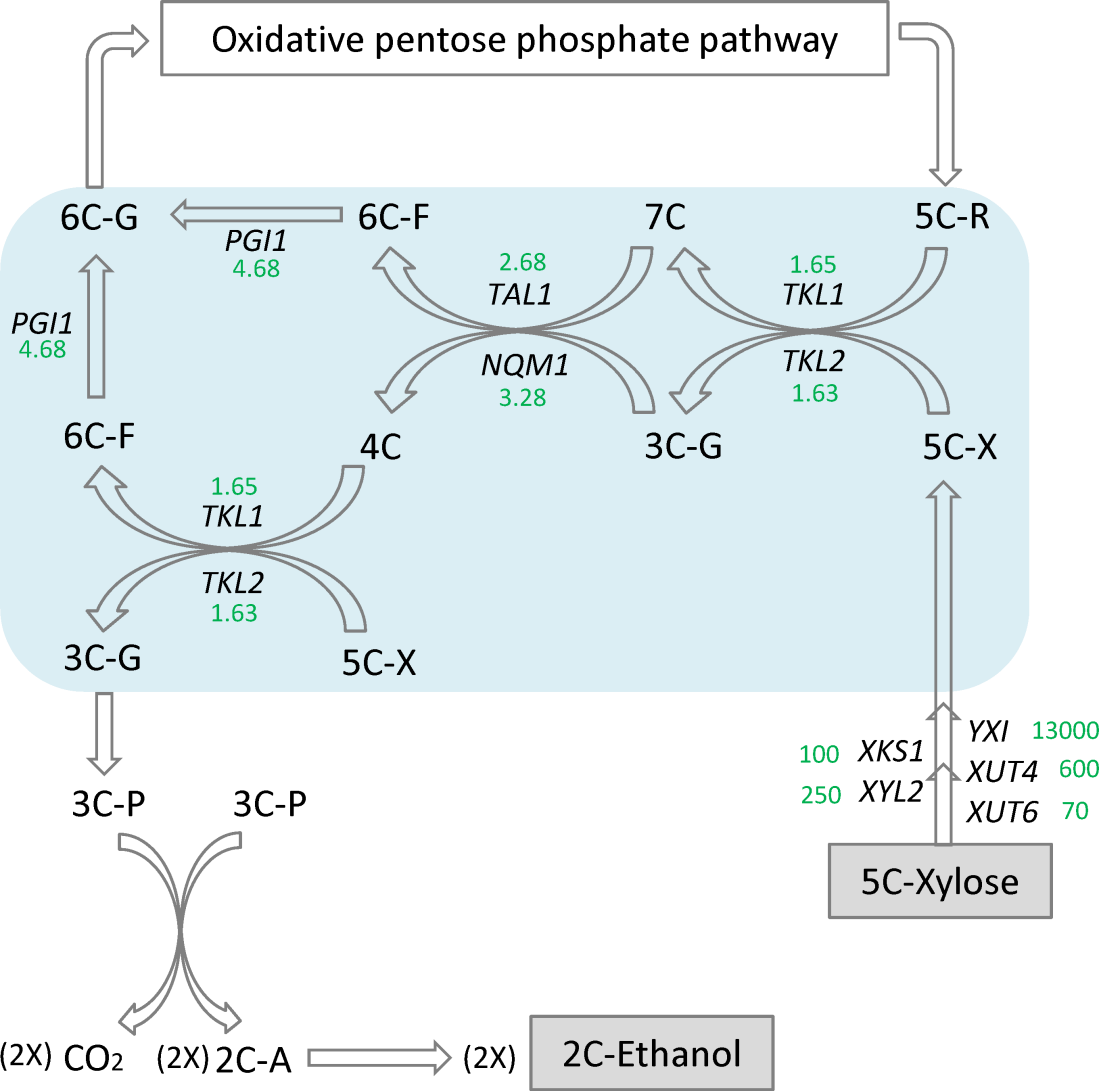
Xylose transformation pathway. A schematic illustration of xylose transformation and metabolism through the non-oxidative pentose phosphate pathway for the genetically engineered industrial yeast *Saccharomyces cerevisiae* NRRL Y-50463. 2C-A stands for acetaldehyde; and 3C-G, glyceraldehydes 3-phosphate; 3C-P, pyruvate; 4C, erythrose 4-phosphate; 5C-R, ribose 5-phosphate; 5C-X, xylulose 5-phosphate; 6C-F, fructose 6-phosphate; 6C-G, glucose 6-phosphate; and 7C, sedoheptulose 7-phosphate. Expression fold changes against the wild type control at 24 h are presented in green.

The second element of the signature expression was concentrated in the non-oxidative pentose phosphate pathway branch involving at least 10 genes. Most genes closely associated with the upper portion of glycolysis were able to maintain a normal level of expression to function in the presence of mixed sugars of glucose and xylose under both aerobic and oxygen-limited conditions (Fig 5). Four genes of *TAL1*, *NQM1*, *TKL1* and *TKL2* were outstanding in the presence of xylose when glucose was completely depleted. Therefore, they are accountable as the most critical genes playing substantial roles for the acquired xylose metabolism in Y-50463 (Fig 6). *TKL1* and *TKL2* encode for transketolases that catalyze conversion of xylulose-5-phosphate and ribose-5-phosphate to sedoheptulose-7-phosphate and glyceraldehyde-3-phosphate [***49, 50***]. *TAL1* and *NQM1* encode transaldolase enzymes that convert sedoheptulose-7-phosphate and glyceraldehydes-3-phosphate to erythrose-4-phosphate and fructose-6-phosphate [***51, 52***]. Overexpression of genes involved in non-oxidative pentose phosphate pathway including *TAL1* and *TKL1* was observed to improve cell growth and the rate of xylose consumption in xylose-utilizing yeast stains [***21, 53–55***]. *TAL1* and *TKL1* were suggested as essential genes for xylose assimilation and utilization [***56***]. A mutation with better xylose fermentation was also found to have elevated protein expression of *TKL1* [***57***]. Our results from this study were consistent with previous observations, and further illustrated these gene interactions and relationships in the non-oxidative pentose phosphate pathway branch. These transketolase and transaldolase genes are actively involved in a serial of sugar transformation reactions through complex interactions to facilitate the efficient metabolism of xylose for the engineered Y-50463 (Fig 6). The enhanced non-oxidative pentose phosphate pathway metabolism drives metabolic flow into the glycolysis. Evidently, functions and interactions between *TAL1-NQM1* and *TKL1-TKL2* are extremely critical for the xylose metabolism in Y-50463 using xylose as a sole source of carbon for ethanol production.

It needs to point out that activation of these genes and interactions are initiated with intermediate xylulose-5-phosphate but not xylose (Fig 6). Expression of *TAL1* did not lead consumption of xylose [***54***]. Therefore, the reduction branch form xylose to xylulose-5-phosphate relies heavily on *YXI*, *XUT4* and *XKS1* (Figs 5 and 6). On the other hand, the active non-oxidative pentose phosphate pathway metabolism also needs intermediate supply of ribose-5-phosphate. In our study, this branch appeared to have an active transcription response.

The third signature expression element included five members of *PRS* gene family with significantly enhanced gene expression response under both aerobic and oxygen-limited conditions. These genes encode 5-phospho-ribosyl-1(alpha)-pyrophosphate synthetases which synthesize PRPP for biosynthesis of nucleotide and many amino acids such as histidine, tryptophan, tyrosine and alanine [***42, 58, 59***]. *PRS* genes are often repressed under fermentation inhibitor challenges associated with declined cell growth [***31***]. The highly activated expression of this group of genes doubtlessly contributed to the sound life cycle and enhanced biosynthesis functions for strain Y-50463. In addition, we also observed that under aerobic conditions, most genes involved in the TCA cycle showed significantly increased expression at almost every step of the reactions at 24 h when xylose was the sole source of carbon supply. This is consistent with observations from another reported flocculating industrial yeast strain [***36***]. For a laboratory yeast strain, a lower level of oxygen enhanced gene expression related to respiratory metabolism under controlled conditions [***60***]. The oxygen-limited condition carried in this study allowed a lower level of oxygen which could lead similar reactions under such conditions for Y-50463. In this study, the current xylose-to-ethanol production by Y-50463 has not reached its maximum theoretical potential yet. Interfere by the uncontrolled endogenous aldose reduction activities appeared exist. Thus, continued efforts of system management are needed for global optimization to improve its efficiency of ethanol production from xylose.

Development of the next-generation biocatalyst remains a continued challenge for efficient utilization of biomass sugars toward a sustainable biofuels production. The industrial yeast strains are more robust and a recent genomic study showed more tolerant signaling pathways of an industrial yeast strain than the model strain S288C [***61***]. Adaptation is a commonly used method for new strain development. The plastic genome of the industrial yeast allows efficient yeast adaptation to varied environmental conditions associated with industrial applications [***31, 62, 63***]. The rate of genome evolution for naturally collected yeast strains was also found to be faster than the laboratory strains [***64***]. Genetically engineered industrial strains have been demonstrated to outperform the laboratory strains for ethanol productivity [***29***]. Their transcription levels of genes involved in xylose metabolism were also found to be higher than the similarly engineered laboratory strains [***65***] The current study and previous reports demonstrated the industrial yeast functions well as a host to engage new gene functions including *YXI* and heterologous xylose transport genes [***28, 33***]. We are confident the industrial yeast, in general, can better serve as a desirable delivery vehicle for development of the next-generation biocatalyst in production of fuels and chemicals from lignocellulose materials.

## Compliance with ethical standards

Authors claim no conflict of interest. This research did not apply any human participants and/or animals. Informed consent was obtained from all individual participants included in the study.

## Supporting information

S1 Table Primers applied for the comparative quantitative gene expression analysis using pathway-based qRT-PCR array assays in this study. (DOCX)

## Abbreviations

2C-A: acetaldehyde
3C-G: glyceraldehydes 3-phosphate
3C-P: pyruvate
4C: erythrose 4-phosphate
5C-R: ribose 5-phosphate
5C-X: xylulose 5-phosphate
6C-F: fructose 6-phosphate
6C-G: glucose 6-phosphate
7C: sedoheptulose 7-phosphate
ATP: adenosine triphosphate
cDNA: complementary deoxyribonucleic acid
Ct: cycle threshold
HMF: 5-(hydroxymethyl)-2-furaldehyde
HPLC: high-performance liquid chromatography
mRNA: messenger ribonucleic acid
NADH: nicotinamide adenine dinucleotide
NADPH: nicotinamide adenine dinucleotide phosphate
OD: optical density
PCR: polymerase chain reaction
PRPP: phosphoribosyl pyrophosphate
qRT-PCR: quantitative real-time polymerase chain reaction
TCA cycle: tricarboxylic acid cycle
XI: xylose isomerase
YXI: yeast xylose isomerase
