# Supplementary material for: Signature pathway expression of xylose utilization in the genetically engineered industrial yeast Saccharomyces cerevisiae
Source: PLoS One. 2018 Apr 5;13(4):e0195633. doi: 10.1371/journal.pone.0195633 (PMC5886582; doi:10.1371/journal.pone.0195633)
Supplement: S1 Table — (DOCX) [file pone.0195633.s001.docx]

**Supporting Table 1. Primers applied for the comparative quantitative gene expression analysis using pathway-based qRT-PCR array assays in this study**

| Primer ID | Sequence 5’-3’ |
| --- | --- |
| ACO1_L | TAACTGGCCATTGGATGTCA |
| ACO1_R | TTTCAAACCATGAGCAGCAG |
| ACO2_L | TCTGTCCCAGCATCCATACA |
| ACO2_R | GCGCAACTCTCCAAGAAATC |
| ACS1_L | TGATGACGCGCTAAGAGAGA |
| ACS1_R | CTGTTGCCCAATCCAAATCT |
| ACS2_L | CTACCGTGCCATTCTTTGGT |
| ACS2_R | ATTGATGGCCATGGTGATTT |
| ADH3_L | AATGGCTGAACGGTTCTTGT |
| ADH3_R | GAATTTTGGCGGCTTGAATA |
| ADH4_L | TGTCACAGCTGGTTTGAAGG |
| ADH4_R | CGATTTCCCCACCGTTAGTA |
| ADH5_L | TGGATGGTACTGGCTTCACA |
| ADH5_L | TCAGCAAGATTGACGTTTGG |
| ADH7_L | ATTTCCAACGCAAAGGATTG |
| ADH7_R | AGATCCGCAGATACCACAGG |
| ALD2_L | TTGTTGGAAATTGCAAGGTG |
| ALD2_R | TGACAACACCAGGTGGAAAA |
| ALD3_L | CCAGGGCTGCTTTTGATAAC |
| ALD3_R | TCTCTAATGCGGCAAGTGTG |
| ALD4_L | AGGCCATTACAAACCATCCA |
| ALD4_R | GATTTACCACCCAGCTCCAA |
| ALD5_L | CGAACAGCCAACAGGGTTAT |
| ALD5_R | CAACGGCTTCATCAACATCA |
| ALD6_L | TGAGCACAGGTTTGAAGGTG |
| ALD6_R | TTGCTTAACACCACCGAATG |
| CDC19_L | TGATGATGTTGAAGCCCGTA |
| CDC19_R | CCTTGAAACCTTGGATGGAA |
| CIT1_L | GGTCGTGCCAATCAAGAAGT |
| CIT1_R | TGCGTTCAAAGTATCCCACA |
| CIT2_L | GCTCAAGGAATTTCCAAGCA |
| CIT2_R | CTTCACCCATTTTGCCATCT |
| CIT3_L | CAGCAATCCCTGGACTTCAT |
| CIT3_R | TCACTCAACGCACTTCCAAC |
| ENO1_L | AAGGTGGTGTTGCTCCAAAC |
| ENO1_R | GCACAGTCCAAACCGATCTT |
| ENO2_L | GCTGACTTGGTTGTCGGTTT |
| ENO2_R | ACAGCCTTGTCACCCAATTC |
| FBA1_L | TGCTTTCGGTAACTGTCACG |
| FBA1_R | CAATGGCTTTTCTTCCTTGC |
| FBP1_L | CCATGGTTGCTGATGTTCAC |
| FBP1_R | CATTGGGAAGGCCTCATAAA |
| FUM1_L | GTCACCGCTTTGAACCCTAA |
| FUM1_R | CCCAATTCCAATGCACTTTC |
| GLK1_L | CTGACGACCTGTTTGGGTTT |
| GLK1_R | AGTGAACCCCAGTTTCATGG |
| GND1_L | TGTCTGCTGATTTCGGTTTG |
| GND1_R | ATGGTCGACCTTGGATTGAG |
| GND2_L | GGTAAGCCATTGGTGGAAAA |
| GND2_R | CTGACAAACAACGAGCGAAA |
| GPM1_L | CGACGCTTCTTCTCCATTCT |
| GPM1_R | TGTCAATGACCAAAGCCAAA |
| GPM2_L | AAGCACACCATCCAATGTCA |
| GPM2_R | TTCGATTAGCTCGGCAGAAT |
| GPM3_L | TCAGCAGACCATGGACGTTA |
| GPM3_R | TGCAACACAGGCATACTTCC |
| HXK1_L | TGCTGTCGACGAACAATCTC |
| HXK1_R | CAACATCAAGCCCTTCTCGT |
| HXK2_L | CCAATTCCATTGGGTTTCAC |
| HXK2_R | GCAACATTGGAACAACATCG |
| IDH1_L | AGAGAAAACACGGAGGGTGA |
| IDH1_R | TCTGGCGATCCTTTCTGTCT |
| IDH2_L | TTCGTCCCGCAAAGTCTATT |
| IDH2_R | GCCAGGGCAAACTATGTGTT |
| IDP1_L | TCAAATGTGCCACCATCACT |
| IDP1_R | CTGTACCGCCGAGAATGTTT |
| IDP2_L | GCTCCTTGGGGTTAATGACA |
| IDP2_R | TGACGTCTCCTTTCCTTGCT |
| IDP3_L | CTTTCAATCCAAGAGCGTGA |
| IDP3_R | TCGGGTGTTATAGTGGCACA |
| KGD1_L | TGGCAAAAAGACCCATCTTC |
| KGD1_R | TACTGGGAGGAGCCTGAAAA |
| KGD2_L | ATTGAGGTCAATTCGCCAGT |
| KGD2_R | ATTCTCCAGAACCCTCAGCA |
| LSC1_L | GATGCTTTTCCCGGTACTGA |
| LSC1_R | CGGCCTTACCACCTATTTCA |
| LSC2_L | TCACTTCGACACCGGTTACA |
| LSC2_R | CCGCTATTCCCGTCTGTTTA |
| MDH1_L | TTGATTTCGCAAACAAACCA |
| MDH1_R | GCCATTGACAACGTAGCAGA |
| MDH2_L | AGGCCAAGAACGGTAAAGGT |
| MDH2_R | TCGCATCTTTTAATGGCACA |
| MDH3_L | GGCCAAGTTTGCTGAAGAAG |
| MDH3_R | CTGCGCTTTCTTACCGTTTT |
| NQM1_L | ATTGGCTGCTTCAAAGTTGG |
| NQM1_R | TATCCATGGCGTTCTCGATT |
| PCK1_L | GGTACCGGGAAAACCACTTT |
| PCK1_R | CTTGGCGTAACAACCACCTT |
| PDA1_L | GTTGCCTGAATCTTCCTTCG |
| PDA1_R | TTGTACAAGGCGTCACAAGC |
| PDB1_L | CCAGACTGCCAACATCATTG |
| PDB1_R | TCTGACGGTCATCGTCTTTG |
| PDC1_L | ACACCATCTTGGCTTTGGTC |
| PDC1_R | CGAAAGCTGGGAATTGAGTC |
| PDC5_L | CACGTTGTTGGTGTTCCATC |
| PDC5_R | TCAGTGATCATGGCAGTGGT |
| PDC6_L | GGAGATTGACCCCAACAAGA |
| PDC6_R | ATACGGCTTTAACCCCCATC |
| PFK1_L | CCAACTGTTGAGCACGAAGA |
| PFK1_R | TTGGTTTCGTTTTCCCAAAG |
| PFK2_L | TCGTGGTGGTCCAGAATACA |
| PFK2_R | TACCTTCGCGCTTCTTGAAT |
| PGI1_L | CTGAAGGTGCCATTTGGAAT |
| PGI1_R | AGCATCGTGGGTAGAAATGG |
| PGK1_L | CTTGCCAGTCGACTTCATCA |
| PGK1_R | CCTTTGCAACAGTAGCAGCA |
| PGM1_L | CCATAGGCGTAACCCTGAAA |
| PGM1_R | TGCTCGCATTCGATATGTTC |
| PGM2_L | TGCTTTGTTCGACGCTAAAA |
| PGM2_R | GTTCTCCGGATGATGCTTGT |
| PGM3_L | TAAGCTAAAGGCCTCCGTTG |
| PGM3_R | CATGATCGTGAGGTGGAATG |
| PRS1_L | TCAGACAAGGTGACCATCCA |
| PRS1_R | TGCAATTCGCTGTCAGATTC |
| PRS2_L | AATGGATCACGGTGCTAAGG |
| PRS2_R | TCGACAATGTCCAAATCCAA |
| PRS3_L | CATGCTTCCCAAATTCAAGG |
| PRS3_R | CCAGCATCTGGCGAAATTAT |
| PRS4_L | CCAAATTGCATCTGCAAGAA |
| PRS4_R | GCAACCAACTTTGCGGTAAT |
| PRS5_L | TGCTCCCATAATTTCCAAGC |
| PRS5_R | TCTTCAACGAGCTCTCAGCA |
| PYC1_L | AACCTGGCAGCAAAAGCTAA |
| PYC1_R | GCCTTAATGATCACCGGGTA |
| PYC2_L | ACCATCTGAATTTGCCAAGC |
| PYC2_R | CCAAATCGTGGGTTCTGACT |
| PYK2_L | AATTGAAATCCTGGCACCTG |
| PYK2_R | TGAATCCAGCATCTGAGTCG |
| RBK1_L | GATACTTTCCTGGGCGGTTT |
| RBK1_R | TACAGTGGCATGCTTTCAGC |
| RKI1_L | CTTTGGAGGATGCCAAGAGA |
| RKI1_R | TTCGGCAACATAAACCACTG |
| RPE1_L | TGGAAGACATGATGCCAAAA |
| RPE1_R | TGGTACCAGCGACAATAACG |
| SDH1_L | AAGAGCCTTTGGTGGTCAGA |
| SDH1_R | TGGCCATAAAGCGTGTGTAA |
| SDH2_L | AGGAAGGCCTTTTGTTTGGT |
| SDH2_R | TTTAGCACTTGGCTCGTCTG |
| SDH3_L | TGAAGAATGTGGCCAGTGAA |
| SDH3_R | TTGTGGCTGGTAAATGGTCA |
| SDH4_L | TCCACTGCAGCTGATTCTTT |
| SDH4_R | CGTACTTGTGCCAAACACCA |
| SFA1_L | ATGTGCAAAAAGGCGATACC |
| SFA1_R | TGGCACCAAATTGAGAACAA |
| SOL1_L | AAAGGCGCCTGTAATCAAGA |
| SOL1_R | GCATCGTCGTCTACAAACCA |
| SOL2_L | CCGATGTTCGACTTGTTCCT |
| SOL2_R | ACCACCCATGCAAGTTTCTC |
| SOL3_L | CTTGCTGAACGAAACAACCA |
| SOL3_R | CTTTGGCGTCTTTCAACACA |
| SOL4_L | TCGTTAGGGGAGCTGCTAAA |
| SOL4_R | TTGGTTCCGACCATTTCATT |
| TAL1_L | ACACTGGTGATTTCGGCTCT |
| TAL1_R | ACTTGGCGTAAGTTGGTTGC |
| TDH1_L | CTCACGCTTCCATCTTCGAT |
| TDH1_R | AAGCCTTGGCAACATATTCG |
| TDH2_L | CGTCGAAGTTGTTGCTTTGA |
| TDH2_L | TGTCATCGTGGGAAACTTCA |
| TDH3_L | GTTGCTTTGAACGACCCATT |
| TDH3_R | GCTTGTCATCGTGGGAAACT |
| THI3_L | ATGTTCCACAGGCTCTGCTT |
| THI3_R | GGAATTCTGGAGTTGGCGTA |
| TKL1_L | AGCCTACGGTTGGGAAGTTT |
| TKL1_R | GCAAGGAACCGTAACCAATG |
| TKL2_L | GTCCAACGCACCAACCTATT |
| TKL2_R | TGGTGTTCGACCAGATTTGA |
| TPI1_L | CTTGAAGGCTTCTGGTGCTT |
| TPI1_R | CGAACTTGGTCTTGTCAGCA |
| XKS1_L | AAGAGAGGTGGCCAAGTTCA |
| XKS1_R | TGTGAGCTTGCGTTTGAATC |
| XUT4_L | ATGCCTGTTTGGAGTTCACC |
| XUT4_R | ACATACAATGGGCTCCTCCA |
| XUT6_L | AAACGATGGTTTGCACTCCT |
| XUT6_R | TTGAGCAACCATCTTGGAGA |
| XYL2_L | AATGGTGCTGGGACATGAAT |
| XYL2_R | CATCTCCGGTGAAAACCTGT |
| YJL045W_L | AGCAGGGATGTTGTTTCCAG |
| YJL045W_R | TATTCCCGGTAGCCTTTCCT |
| YXI_L | AACATTACCGACCCAATGGA |
| YXI_R | CACCTTCTGGAGCAATGTCA |
| ZWF1_L | CCAGTTTTTGAATGCCTCGT |
| ZWF1_R | ACAGATGGTTCTGCATCACG |
